# Avian Influenza A(H5N1) Virus in Egypt

**DOI:** 10.3201/eid2203.150593

**Published:** 2016-03

**Authors:** Ghazi Kayali, Ahmed Kandeil, Rabeh El-Shesheny, Ahmed S. Kayed, Asmaa M. Maatouq, Zhipeng Cai, Pamela P. McKenzie, Richard J. Webby, Samir El Refaey, Amr Kandeel, Mohamed A. Ali

**Affiliations:** St. Jude Children’s Research Hospital, Memphis, Tennessee, USA (G. Kayali, P.P. McKenzie, R.J. Webby);; National Research Centre, Giza, Egypt (A. Kandeil, R. El-Shesheny, A.S. Kayed, A.M. Maatouq, M.A. Ali);; Georgia State University, Atlanta, Georgia, USA (Z. Cai);; Ministry of Health and Population, Cairo, Egypt (S. El Refaey, A. Kandeel)

**Keywords:** avian influenza, subtype H5N1, surveillance, vaccination, respiratory infections, vector-borne infections, viruses, influenza, Egypt

## Abstract

In Egypt, avian influenza A subtype H5N1 and H9N2 viruses are enzootic in poultry. The control plan devised by veterinary authorities in Egypt to prevent infections in poultry focused mainly on vaccination and ultimately failed. Recently, widespread H5N1 infections in poultry and a substantial increase in the number of human cases of H5N1 infection were observed. We summarize surveillance data from 2009 through 2014 and show that avian influenza viruses are established in poultry in Egypt and are continuously evolving genetically and antigenically. We also discuss the epidemiology of human infection with avian influenza in Egypt and describe how the true burden of disease is underestimated. We discuss the failures of relying on vaccinating poultry as the sole intervention tool. We conclude by highlighting the key components that need to be included in a new strategy to control avian influenza infections in poultry and humans in Egypt.

## Abstract 2

An aggressive plan to curb these infections in poultry is urgently needed.

An unprecedented increase in the number of human infections with the highly pathogenic avian influenza A(H5N1) virus was observed in Egypt during the 2014–15 winter season. The World Health Organization reported that 31 cases were confirmed in 2014, of which 27 were in persons infected as of September (*1*). The Ministry of Health and Population in Egypt confirmed 31 cases in 2014 and 88 in January and February 2015. Thus, the official number of cases during September 2014–February 2015 was 114, including 36 deaths. Furthermore, in February 2015, the first human case of subtype H9N2 virus infection in Egypt was reported. These events compelled national and international authorities to examine the reasons behind the increase in human infections and implement control measures.

In Egypt, highly pathogenic avian influenza subtype H5N1 virus was first reported in poultry in 2006 and was declared to be enzootic in 2008 (*2*,*3*). As an initial response, the government of Egypt devised a comprehensive response plan that included increasing awareness, culling infected poultry, zoning and movement restrictions, and emergency vaccination of parent flocks (*3*,*4*). However, the virus continued to circulate, and infections were reported in more governorates. The authorities then decided to increase vaccination to cover all commercial flocks and backyard poultry (*3*). Eventually, vaccination became the only tool used to control H5N1 virus in Egypt, as other aspects of the control plan became neglected. This strategy failed to control the spread of H5N1 virus, given that outbreaks in poultry continued to occur.

The inadequate control measures enabled H5N1 viruses to mutate. Genetic drift in the hemagglutinin (HA) gene was observed each year and was more profound after 2008, when the virus was declared enzootic (*4*–*6*). Two subclades of H5N1 viruses, 2.2.1 and 2.2.1.1, co-circulated in poultry from late 2009 through 2011 (*5*–*7*). Subclade 2.2.1.1 viruses are thought to have emerged as escape mutants because of vaccine pressure (*8*). As of 2012, subclade 2.2.1.1 viruses were rarely detected, but subclade 2.2.1 viruses continued to evolve to form a new phylogenetic cluster (*5*). Subclade 2.2.1 and 2.2.1.1 viruses were also antigenically distinct (*9*–*11*).

Most human cases of H5N1 infection in Egypt were caused by infection with subclade 2.2.1 H5N1 viruses, which are abundant in backyard poultry (*12*). Epidemiologic analysis of human H5N1 cases reported during 2006–2010 showed that the case-fatality rate was 34% and differed significantly by sex (higher among female patients), age (increased with age), and time to hospitalization (decreased with faster hospitalization) (*4*,*13*). By 2015, most of the reported H5N1 human cases worldwide were in Egypt (37%, 292/784) (*1*).

This increase in human infections and the continuous circulation of H5N1 and H9N2 viruses in poultry in Egypt have raised concerns for public health and animal health. Here we analyze the current situation of H5N1 viruses in Egypt. We discuss the evolution of the viruses in poultry, describe the epidemiology of human infection, analyze the effect of poultry vaccination, and provide insight on how to move forward for controlling H5N1 virus circulation in poultry.

## Avian Influenza in Poultry in Egypt

### Surveillance

In Egypt, a systematic surveillance program for avian influenza viruses has been in place since 2009. The program, a collaborative effort between the Center of Scientific Excellence for Influenza Viruses in Egypt and the St. Jude Center for Excellence for Influenza Research and Surveillance in the United States, provided an important tool for understanding the ecology of avian influenza viruses in poultry in Egypt. Commercial and semicommercial farms, abattoirs, backyard flocks, and live bird markets located in different governorates are sampled on a monthly basis regardless of the presence of disease symptoms in poultry. In each governorate, the same locations are continuously sampled by a poultry veterinarian, who collects 1 swab sample per bird; depending on the size of the poultry population, as many as 5 birds are sampled per flock. Birds are not randomly selected, and samples also are collected from sick or dead birds found onsite. Samples are mostly from chickens, but ducks, geese, turkeys, pigeons, and quails also are sampled. This program enabled detection of genetic and antigenic changes in H5N1 viruses in Egypt. It provided important epizootiologic data and described the poultry sectors acting as reservoirs of H5N1 viruses. 

**Figure 1:**
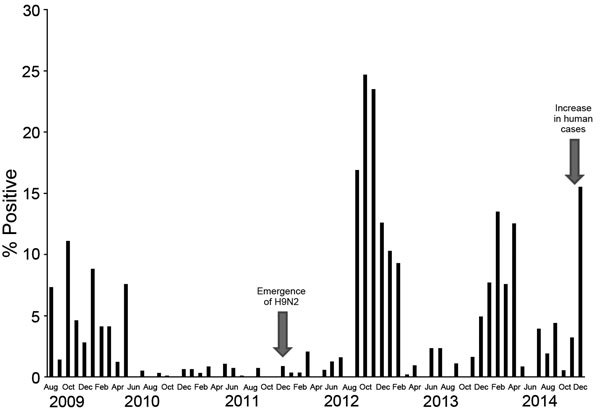
Monthly positivity rate of poultry infection with avian influenza viruses (all types), Egypt, August 2010–December 2014. A seasonal pattern is shown by sharp increases in rates during colder months (November–March). Emergence of H9N2 virus in poultry and an increase in human H5N1 cases are indicated.

The rate of avian influenza infection during August 2009–July 2010 was 5%, was exclusively attributable to H5N1 infection, and was more concentrated in the commercial production sector (*14*). From August 2010 through January 2013, the positivity rate increased to 10%, and the infection was detected in all poultry production sectors (*6*). In 2011, H9N2 viruses emerged and were detected by this program and other surveillance activities in Egypt (*6*,*15*,*16*). The monthly positivity rate of avian influenza infection from August 2009 through December 2014 (Figure 1) showed that avian influenza infection in poultry follows a seasonal pattern, with sharp increases during the colder months (November through March). After the detection of H9N2 virus in 2011, the positivity rate during colder months was higher than that in the period when H5N1 was the only virus infecting poultry, exceeding 20% in some months. Furthermore, co-infection with H5N1 and H9N2 viruses was frequently detected (*6*).

**Figure 2:**
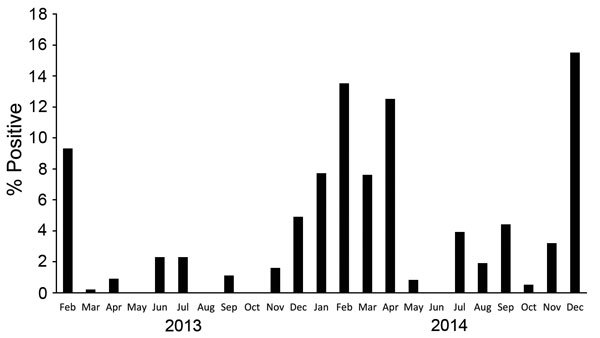
Monthly positivity rate of infection with avian influenza viruses (all types), Egypt, February 2013–December 2014. As in Figure 1, a seasonal pattern is shown by sharp increases in rates during colder months (November–March).

**Figure 3:**
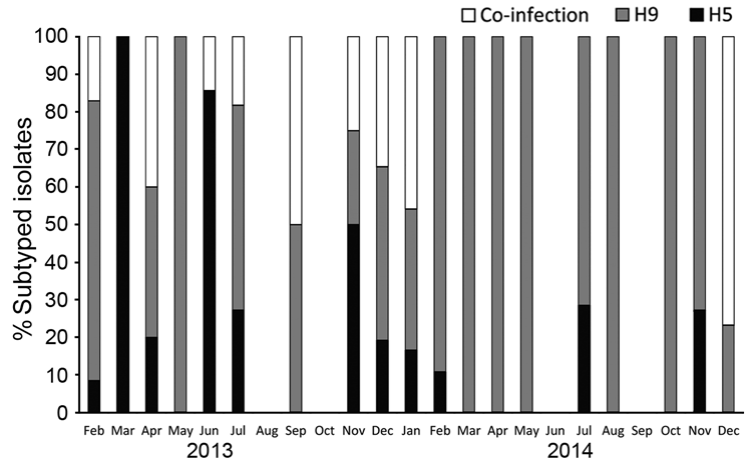
Subtypes of influenza A viruses detected in poultry by using reverse transcription PCR, by month, Egypt, February 2013–December 2014.

We further analyzed data collected through the surveillance program during February 2013–December 2014, when 4,858 cloacal and 3,049 oropharyngeal samples were collected (range 120–700 samples monthly). The positivity rate for any avian influenza infection was 4.7%. A higher rate of infection was observed in oropharyngeal swab samples. Detection rates differed significantly by governorate, species, and poultry production sector. No significant differences by the birds’ health status or age were observed (Table 1). The same seasonal pattern observed during August 2009–July 2010 (Figure 1) was observed during this period (Figure 2). H9N2 virus was more frequently detected as a single virus causing infection or as co-infecting the same bird with H5N1 virus (Figure 3). Both H5N1 and H9N2 viruses were circulating in the more recent surveillance months, when more human cases were reported.

**Table 1:** Epizootiologic data on avian influenza viruses (all types), Egypt, February 2013–December 2014*

Variable	Collected samples, no. (%)†	Influenza A–positive samples, no. (%)‡	p value§
Sample type			
Cloacal	4,858 (61.4)	112 (2.3)	<0.01
Oropharyngeal	3,049 (38.6)	234 (7.7)	
Governorate			
Cairo	1,116 (14.1)	9 (0.8)	<0.01
Daqhaliya	2,031 (25.7)	136 (6.7)	
Qalubiya	809 (10.2)	22 (2.7)	
Menofiya	13 (0.2)	0	
Sharqiya	2,160 (27.3)	123 (5.7)	
Fayyoum	1,642 (20.8)	48 (2.9)	
BeniSuef	30 (0.4)	4 (13.3)	
Asiut	69 (0.9)	4 (5.9)	
El Minya	38 (0.5)	0 (0)	
Species			
Chickens	6,863 (86.8)	322 (4.7)	0.01
Ducks	606 (7.7)	15 (2.5)	
Geese	58 (0.7)	0	
Pigeons	243 (3.1)	2 (0.8)	
Turkey	57 (0.7)	1 (1.8)	
Quail	80 (1.0)	6 (7.5)	
Location			
Abattoir	150 (1.9)	0	0.01
Commercial farm	4,359 (55.1)	200 (4.6)	
Backyard flock	1,678 (21.2)	61 (3.6)	
Live bird market	1,720 (21.8)	85 (4.6)	
Bird health status			
Healthy	5,799 (73.3)	238 (4.1)	NS
Ill	1,629 (20.6)	86 (5.3)	
Dead	479 (6.1)	22 (4.6)	
Age group, y			
0–1	7,819 (98.9)	342 (4.4)	NS
>1	88 (1.1)	4 (4.5)	


*NS, not significant. †Percentage of total samples collected. ‡Of samples in category. §By χ^2^ test comparing positivity rates across variable categories.

### Genetic Analysis

**Figure 4:**
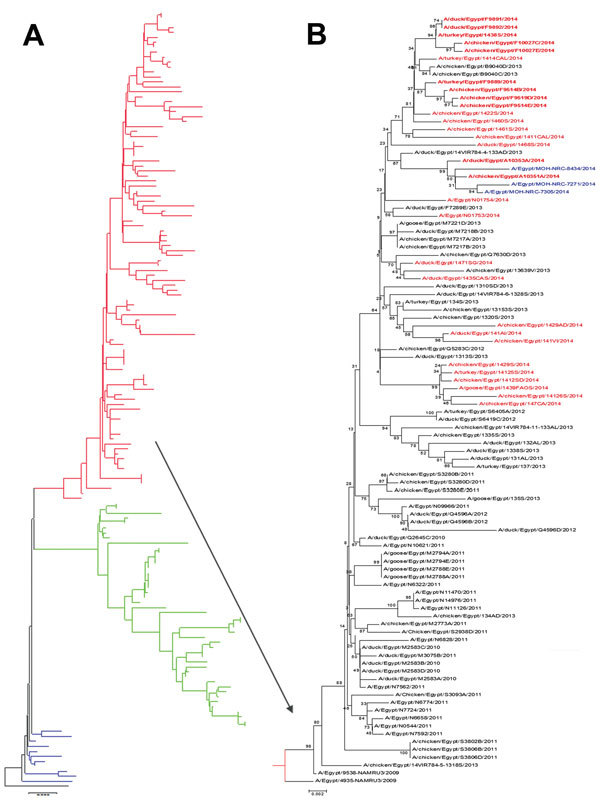
Phylogenetic tree of the hemagglutinin genes of avian influenza subtype H5N1 viruses isolated in Egypt during 2006–2014 and reference isolates from GenBank. Phylogenetic analysis was conducted by using the neighbor-joining algorithm with the Kimura 2-parameter model. Strain A/bar-headed goose/Qinghai/3/2005 was used as the root for the tree, and the reliability of phylogenetic inference at each branch node was estimated by the bootstrap method with 1,000 replications. Evolutionary analysis was conducted by using MEGA6 (http://www.megasoftware.net). A) Clade 2.2 viruses from 2006–2008 are shown in blue, subclade 2.2.1.1 viruses are shown in green, and clade 2.2.1 viruses are shown in red. B) Human viruses sequenced for this study are shown in blue. Boldface red font indicates avian viruses isolated in 2014 and sequenced for this study; lightface red font indicates other viruses from GenBank. *Indicates that 2014 viruses were grouped in 1 lineage. Scale bars indicate nucleotide substitutions per site.

Phylogenetic analysis of all the HA genes of H5N1 viruses in Egypt available in GenBank shows considerable evolution over time (Figure 4). H5N1 was first detected in 2006 and remained relatively stable until 2008. In 2008, subclade 2.2.1.1 emerged and continued in circulation until early 2011. At the same time, clade 2.2.1 viruses also were in circulation. Within this group, further drift was observed, and as of 2011, viruses grouped together and formed a new cluster characterized by a set of mutations (*5*). Viruses from late 2013 and 2014 branched together within this new cluster. Some avian H5N1 viruses in 2014 possessed mutations R140K in antigenic site A and A86V in antigenic site E of the HA gene, similar to other viruses in the new cluster. The new cluster was classified as clade 2.2.1.2 (*17*).

Phylogenetic analysis of the neuraminidase (NA) gene and the internal genes showed a similar pattern of evolution as that for the HA gene (Technical Appendix). Currently circulating viruses belong to the new cluster. No major mutations were observed in viruses circulating during 2013–2014.

Genetic analysis of the H9N2 viruses in poultry in Egypt showed that those viruses belonged to the G1 lineage. Also, they possessed several genetic markers of increased transmission to mammalian hosts (*18*).

### Antigenic Analysis

**Figure 5:**
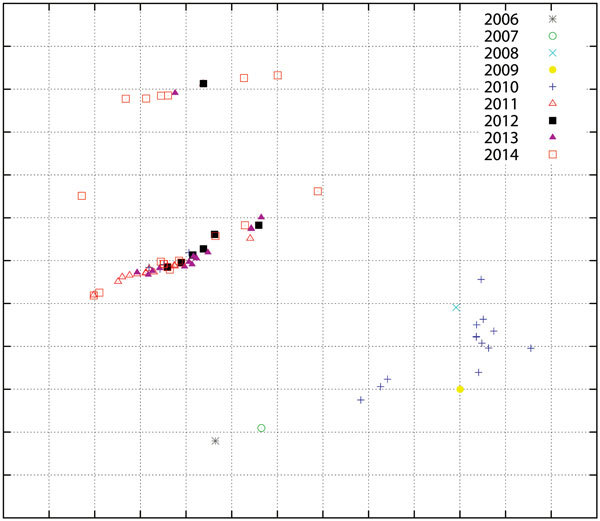
Antigenic cartography of reactivity of highly pathogenic avian influenza A(H5N1) virus isolates from Egypt, 2006–2014. The map was produced by using hemagglutination inhibition assay data generated with a panel of monoclonal antibodies and by using AntigenMap (http://sysbio.cvm.msstate.edu/AntigenMap). One unit (grid) represents a 2-fold change in the assay results. Each mark on the map represents results for 1 isolate.

The genetic drift of H5N1 viruses in Egypt led to antigenic variability. When tested against a panel of H5N1 virus monoclonal antibodies, subclades 2.2.1 and 2.2.1.1 are antigenically distinct (*6**,**10**,**11*). A more recent analysis revealed that several strains from 2013 and 2014, especially those with the R140K mutation in the HA gene, had a distinct antigenic composition compared with other viruses of the new cluster (Figure 5).

## Human Infection with H5N1 Viruses

### Human Cases

**Figure 6:**
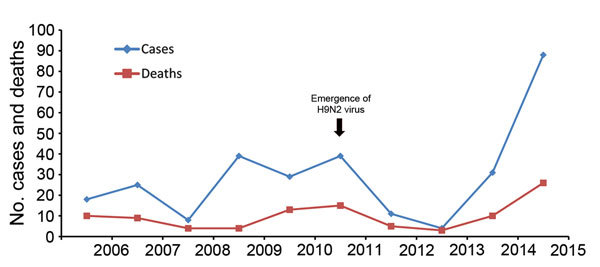
Human cases of avian influenza A(H5N1) virus infection and associated deaths, Egypt, 2006–2015. Data for 2015 include cases confirmed in January and February only. For reference, the emergence of H9N2 virus in poultry is shown (arrow).

During 2006–2015, the estimated number of confirmed human cases of H5N1 infection in Egypt was 292, with a 34% case-fatality rate. The number of reported cases by year for 2006–2015 (Figure 6) shows that, before the 2014–15 winter season, the annual number of cases never exceeded 40. However, in just 2 months, January and February 2015, a total of 88 cases were reported. The most recent cases occurred in persons who reported exposure to backyard poultry (70%), bred domestic birds (26%), slaughtered poultry (14%), or were exposed to dead birds (4%). The main clinical signs and symptoms were fever (98%), sore throat (94%), and cough (83%). Overall, the case-fatality rate was lower than the 67% calculated for human cases globally, excluding those from Egypt. Within Egypt, the case-fatality rate annually ranged from 10% to 75%.

### Characterization of Recent Human Viruses

We sequenced the full genomes of 2 H5N1 viruses isolated from infected humans in Egypt during November 2014 (A/Egypt/MOH-NRC-7271/2014 and A/Egypt/MOH-NRC-7305/2014; GenBank accession nos. KP7022162–KP7022177). We also sequenced the HA segment of a third virus, A/Egypt/MOH-NRC-8434/2014 (GenBank accession no. KR063683.1), isolated from a human in December 2014. In addition, we sequenced 2 poultry viruses (A/duck/Egypt/A10353A/2014 and A/chicken/Egypt/A10351A/2014) obtained from the same places around the same time the human cases were detected (Figure 4). The poultry and human viruses branched together. The strains of human influenza viruses from Egypt carried several mutations that were novel or rare in previously circulating strains and have not been characterized before. Two viruses had mutations M66I, I529V, and E249K in the polymerase basic 2 gene. These mutations were present in 1 virus isolated from a chicken in November 2014. Mutations M66I and I529V were previously seen in chicken viruses from January 2014. Mutation I529V was previously seen in a single chicken isolate from 2013.

Sequencing results for the 2 human viruses isolated in November 2014 for which the full genome sequences were obtained showed the presence of mutation G22E in the polymerase basic 1–frameshift 2 (PB1-F2) gene; the mutation was not found in other viruses. Sequencing results also showed 3 unique mutations in the polymerase protein of the 2 viruses: L342M, E351D, and F708L. These mutations were also seen in a chicken virus obtained during the same month that the humans became ill, and mutations L342M and E351D were previously seen in chicken viruses isolated in January 2014. Mutation K373R was common in the HA protein of all 3 human viruses and was previously observed in a chicken virus from the same period and in 2 human viruses isolated in 2009. The NA protein of the 2 fully sequenced human viruses had novel mutations V43I, I94V, V264I, and V304I, which were also present in the chicken isolate of the same period. One novel mutation, R452K, was observed in the nucleoprotein gene of the 2 fully sequenced viruses; this mutation was also seen in the chicken isolate of the same period, 2 chicken isolates from January 2014, and 1 chicken isolate from 2013.

The results of our genetic analysis indicate that the viruses infecting humans in November and December 2014 had a genetic composition almost exactly the same as that of the avian viruses circulating at that time. These human and avian viruses had a set of mutations throughout the genome that places the viruses on the same phylogenetic branch (Technical Appendix). The role of these mutations, whether individually or in combination, is not known. Determining whether the mutations we found contributed to the recent increase in human H5N1 infections in Egypt would require basic science research that might fall under the “gain of function” category, in which viruses are genetically manipulated under laboratory conditions to study their effects on mammalian hosts.

### Extent of Avian Influenza Infection in Egypt

In Egypt, the number of reported human cases of avian influenza infection appears to be underestimated. An underestimation might result in an overestimation of the case-fatality rate, but it would certainly underestimate the extent of human infection with avian influenza viruses. Results from a controlled, serologic cohort study of persons in Egypt exposed and not exposed to poultry estimated the seroprevalence of antibodies against H5N1 (titers >80) at 2% (*19*). If this seroprevalence were to be extrapolated to the entire poultry-exposed population in Egypt, the true number of infections would amount to several hundred thousand. These figures are even more striking when it comes to human infection with H9N2 viruses. The seroprevalence of H9N2 antibodies detected in the same cohort study (*19*) ranged from 5.6% to 7.5%, whereas just 1 case of H9N2 infection was reported.

H5N1 viruses elicit a poor humoral immune response, providing low antibody titers that typically fade over a short period (*20*,*21*). Thus, relying on serologic testing to detect prevalence or incidence of infection can yield underestimated results. This outcome was evident when we used a microneutralization assay to test serum samples from 38 contacts of persons with confirmed H5N1 infection; no antibodies against H5N1 virus were found, but 5 contacts had low levels (<1:20) of antibodies against H9N2 virus. This finding suggests that the extent of avian influenza infection in humans is even higher than what is currently thought. Human genetic predisposition to infection with avian influenza viruses is an important epidemiologic question that is not well studied, although some reports suggest that genetics play a role in susceptibility to infection (*22*,*23*). Hence, estimating the true incidence of human infection with avian influenza viruses and determining the accompanying risk factors need further study.

## H5 Influenza Vaccines for Poultry

**Figure 7:**
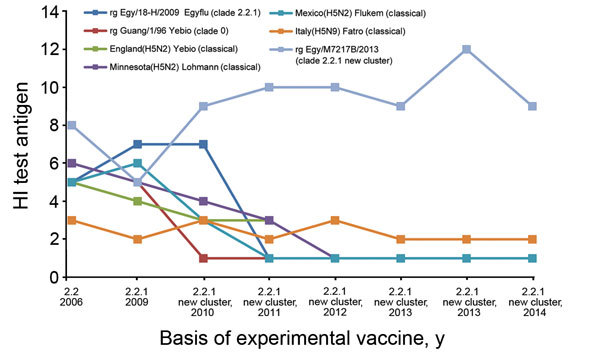
Cross-reactivity of antisera raised against commercial and experimental inactivated H5 vaccines against avian influenza A(H5N1) virus isolates from Egypt, 2006–2014. Antisera from chickens immunized with the H5 vaccines were tested by using a hemagglutination inhibition (HI) assay against virus isolates from Egypt during 2006–2014 (x-axis). Egy, Egypt; Guang, Guangdong; rg, reverse genetics–engineered reassortant.

As of 2006, at least 24 commercial inactivated avian influenza H5 vaccines were licensed for use at poultry farms in Egypt (Table 2). Different viruses were used as vaccine seed strains, including classical H5 lineage viruses and reverse genetics–engineered reassortant viruses containing H5N1 virus HA and NA genes and the remaining genes from A/Puerto Rico/8/1934(H1N1). Farm owners decide which vaccine to use, if any. Amino acid sequence similarities between vaccine strains and the consensus sequence of H5N1 isolates circulating in Egypt during 2013–2014 ranged from 84.0% to 99.6% of A/chicken/Mexico/232/94 (H5N2) and reverse genetics–engineered A/chicken/Egypt/M2583D/2010 (H5N1), respectively. Serum samples obtained from chickens vaccinated with commercial vaccines or an experimental vaccine based on clade 2.2.1 A/chicken/Egypt/M7217B/2013(H5N1) were tested against H5N1 viruses isolated in Egypt during 2006–2014 (Figure 7). Commercial vaccines showed variable reactivity against earlier antigens, but reactivity declined as the virus mutated. The experimental vaccine was highly reactive with all antigens, especially for more recent viruses. The genetic dissimilarity and poor reactivity between commercial vaccines and currently circulating viruses indicate that the vaccines are not efficacious in the field. These vaccines confer partial protection and thus might lead to vaccine-induced escape mutants, thereby complicating, rather than solving, the problem of H5N1 virus circulation in Egypt. Previous reports have indicated that improper antigenic matching between vaccines and circulating viruses might reduce vaccine efficacy (*24*–*27*).

**Table 2:** H5 commercial inactivated oil-emulsion vaccines used for immunization of poultry against avian influenza A(H5N1) virus, Egypt, 2006

Vaccine trade name	Virus used	Lineage	Sequence similarity, %	Manufacturer, city, country
AI-VAC H5	A/chicken/Italy/22A/1998(H5N9)	Classical	90.7	FATRO, Ozzano dell'Emilia, Italy
CEVAC FLUKEM	A/chicken/Mexico/232/1994(H5N2)	Classical	84	Ceva, Mexico City, Mexico
VOLVAC IV KV	A/chicken/Mexico/232/1994(H5N2)	Classical	84	Boehringer Ingelheim, Ingelheim am Rhein, Germany
AIV Vaccine	A/turkey/Engl and /N28/1973(H5N2)	Classical	91.4	Yebio, Qingdao, China
AIV Vaccine	A/turkey/Minnesota/3689–1551/1981(H5N2)	Classical	89.8	Lohmann, Waterville, Maine, United States
Nobilis Influenza H5N2	A/duck/Potsdam/1402/1986(H5N2)	Classical	91.6	Merck, Kenilworth, New Jersey, United States
Optimune AIV	A/turkey/Wisconsin/1968(H5N9)	Classical	88.3	Ceva Biomune, Mexico City, Mexico
Avian Influenza H5	A/chicken/Mexico/232/1994(H5N2)	Classical	84	Avimex Animal Health Mexico City, Mexico
VOLVAC IV +ND KV	A/chicken/Mexico/232/94(H5N2) and Lasot and V	Classical	84	Boehringer Ingelheim, Ingelheim am Rhein, Germany
CEVAC NEW FLU-KEM	A/chicken/Mexico/232/94(H5N2) and Lasot and V	Classical	84	Ceva, Mexico City, Mexico
ITA FLU	A/chicken/Mexico/232/1994(H5N2)	Classical	84	Laprovet, Notre-Dame-d’Oé, France
Reassortant AIV (subtype H5N1) Vaccine (strain Re-1)	RGA/goose/Guangdong/1996(H5N1)(Re-1)	Clade 0	93.8	Zhaoqing DaHuaNong Biology Medicine, Sihui, China
Reassortant AIV (Subtype H5N1) Vaccine (srain Re-1)	RGA/goose/Guangdong/1996(H5N1)(Re-1)	Clade 0	93.8	Yebio, Qingdao, China
PoulvacFluFend H5N3 RG	RGA/chicken/VN/C58/2004(H5N3)	Clade 1	95.1	Fort Dodge Animal Health, Fort Dodge, Iowa, USA
MEFLUVAC	RGA/chicken/Egypt/Q1995D/2010(H5N1) and RGA/chicken/Egypt/M2583D/2010(H5N1)	Clade 2.2	93.8 and 99.6	ME-VAC, Cairo, Egypt
SER-VACC FLU	RGA/chicken/Egypt/M2583D/2010(H5N1)	Clade 2.2	99.6	Veterinary Serum and Vaccine Institute, Cairo, Egypt
ME FLUVAC H5+H9	RGA/chicken/Egypt/Q1995D/2010(H5N1) and A/chicken/Egypt/114940v/NLQP/2011(H9N2)	Clade 2.2	93.8	ME-VAC, Cairo, Egypt
ME FLUVAC One	RGA/duck/Egypt/M2583D/2010(H5N1)	Clade 2.2	99.6	ME-VAC, Cairo, Egypt
ME FLUVACH5+ ND	RGA/chicken/Egypt/Q1995D/2010(H5N1) and NDV/chicken/Egypt/11478AF/2011(ND)	Clade 2.2	93.8	ME-VAC, Cairo, Egypt
ME FLUVAC Super H5 +H9+ ND	RGA/chicken/Egypt/Q1995D/2010(H5N1), RGA/duck/Egypt/M2583D/2010(H5N1), A/chicken/Egypt/114940v/NLQP/2011(H9N2), and NDV/chicken/Egypt/11478AF/2011(ND)	Clade 2.2	93.8 and 99.6	ME-VAC, Cairo, Egypt
Egy FLU	RGA/chicken/Egypt/18-H/2009(H5N1)	Clade 2.2	94.9	Harbin Veterinary Research Institute, Harbin, China
Inactivated Reassortant Avian Influenza Virus Vaccine (H5N1 Subtype, Re-6 Strain)	RGA/duck/Guangdong/S1322/2006(H5N1)(Re-6)	Clade 2.3.2	Not performed	Yebio, Qingdao, China
Reassortant AIV (Subtype H5N1) Vaccine (Strain Re-5)	RGA/duck/Anhui/1/2006(H5N1)(Re-5)	Clade 2.3.4	94.9	Merial, Duluth, Georgia, United States
Reassortant AIV (Subtype H5N1) Vaccine (Strain Re-5)	RGA/duck/Anhui/1/2006(H5N1)(Re-5)	Clade 2.3.4	94.9	QYH, Beijing, China


All vaccines used in Egypt are licensed by the Ministry of Agriculture and Land Reclamation on the basis of laboratory evaluation results. For influenza virus vaccines, this evaluation involves vaccinating poultry and challenging them with an H5N1 virus. Until recently, the challenge virus was a 2008 H5N1 virus isolate, A/chicken/Egypt/1709-6/2008(H5N1) (GenBank accession no. EU 717857). Against the 2008 isolate, those vaccines were efficacious in the laboratory setting but not in the field because the circulating viruses were not antigenically matched to the vaccine seed strains, highlighting the failure of the only tool used to control avian influenza among poultry in Egypt. 

## Proposed Avian Influenza Control Plan

The avian influenza situation in Egypt is deteriorating, evident in the fact that H5N1 and H9N2 viruses are enzootic in poultry and that incidence of H5N1 and H9N2 infection is increasing. The problem is also evident in the sharp increase in human H5N1 cases and the detection of the first human H9N2 case. Thus, it is imperative that authorities in Egypt devise and implement an aggressive control plan to curb the spread of disease in human and animal populations. The control plan must include the following elements: 1) mapping of the unlicensed, small-scale poultry farms that have become abundant in rural areas; 2) increasing the biosecurity levels of these small farms by using inexpensive tools; 3) revamping veterinary and public health surveillance and conducting joint human–animal interface surveillance and risk-assessment exercises; 4) encouraging poultry owners to report outbreaks and providing them appropriate compensation; 5) intervening, when poultry outbreaks are reported, by culling infected poultry and setting monitoring zones around each focus point; 6) properly decontaminating infected farms; 7) encouraging the use of disinfectants in backyards where poultry are raised; 8) increasing awareness about the effects of avian influenza; 9) testing patients with suspected influenza forH5N1 and H9N2 virus; and 10) reevaluating the vaccination strategy, including that for H9N2 virus. If vaccination is to remain an important tool in the control plan, then the following aspects should be considered: 1) matching vaccine strains to currently circulating strains; 2) matching challenge strains to currently circulating strains; 3) maintaining high vaccination coverage; 4) ensuring vaccine efficacy not only in a laboratory setting but also in the field; and 5) evaluating vaccine efficacy on an annual basis.

## Conclusions

Egypt is one of the few countries where H5N1 virus has become enzootic and is the only country with a high number of H5N1 outbreaks among poultry and cases among human. During the 2014–15 winter season, a sudden and substantial increase in human infection with H5N1 viruses was observed. There is no obvious or confirmed reason for this increase, but data indicate the following: 1) H9N2 virus is co-circulating and co-infecting with H5N1 viruses, 2) H5N1 viruses causing the infections possess some mutations that were rarely seen in the past, and 3) the poultry vaccination program is failing. However, our perspective was limited to the data available through our surveillance program, which might not be representative of the epizootiology of avian influenza virus in Egypt. Regardless of the causes of the recent increase in human H5N1 cases, this situation evolved because of the ineffective control strategy that was implemented. Controlling the situation requires a One Health approach, but certainly the greater share of responsibility now lies with the veterinary side. 

**Technical Appendix.** Phylogenetic trees of the neuraminidase gene and internal genes of avian influenza A(H5N1) viruses in Egypt, 2013–2014.
