# Supplementary material for: Avian Influenza A(H5N1) Virus in Egypt
Source: Emerg Infect Dis. 2016 Mar;22(3):379–88. doi: 10.3201/eid2203.150593 (PMC4766899; doi:10.3201/eid2203.150593)
Supplement: Supplementary file 1 — Technical Appendix. Phylogenetic trees of the neuraminidase gene and internal genes of avian influenza A(H5N1) viruses in Egypt, 2013–2014. [file 15-0593-Techapp-s1.pdf]

Article DOI: http://dx.doi.org/10.3201/eid2203.150593

Avian Influenza A(H5N1) Virus in Egypt

Technical Appendix

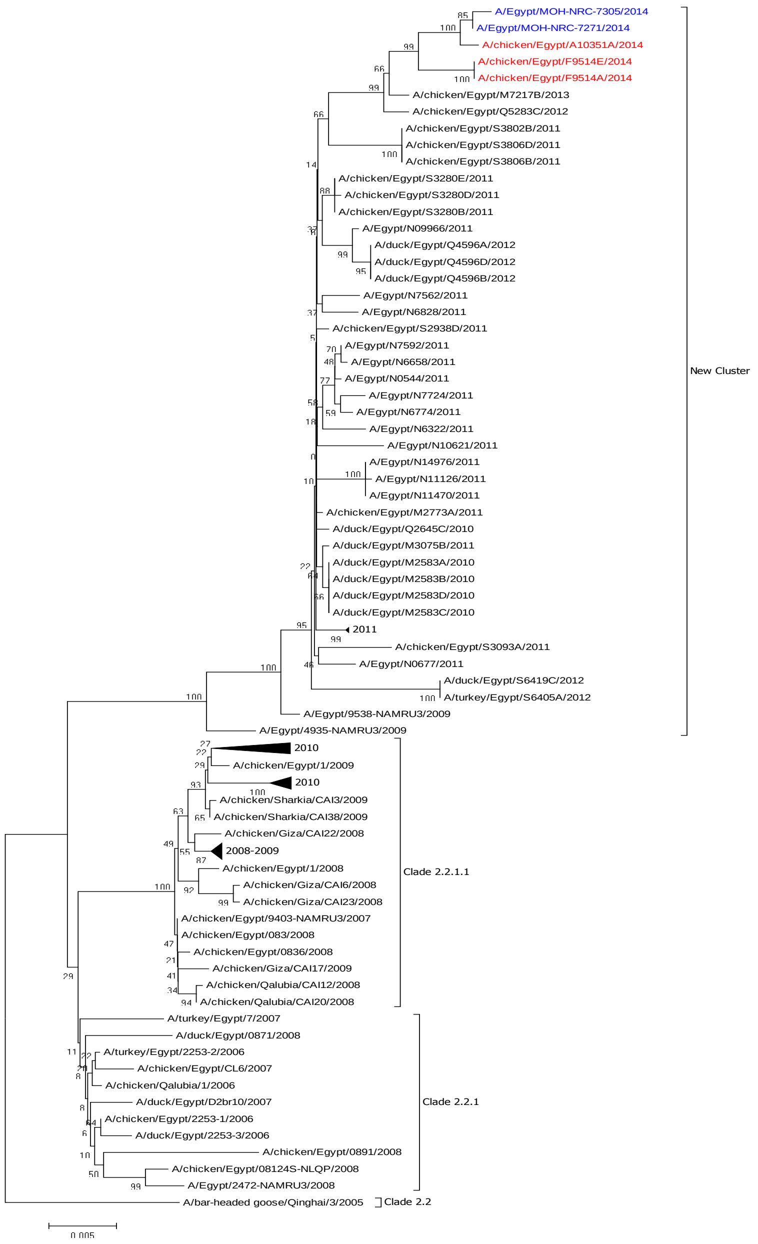

Technical Appendix Figure 1. Phylogenetic tree of the PB2 gene. Phylogenetic analysis was done using the neighbor-joining algorithm with the Kimura 2-parameter model. A/bar-headed goose/Qinghai/3/2005 was used as the root for the tree and the reliability of phylogenetic inference at each branch node was estimated by the bootstrap method with 1,000 replications, evolutionary analysis were conducted in MEGA6. Human viruses sequenced for this study are marked with a blue color. Avian viruses were isolated in 2014 and sequenced for this paper are marked with a red color.

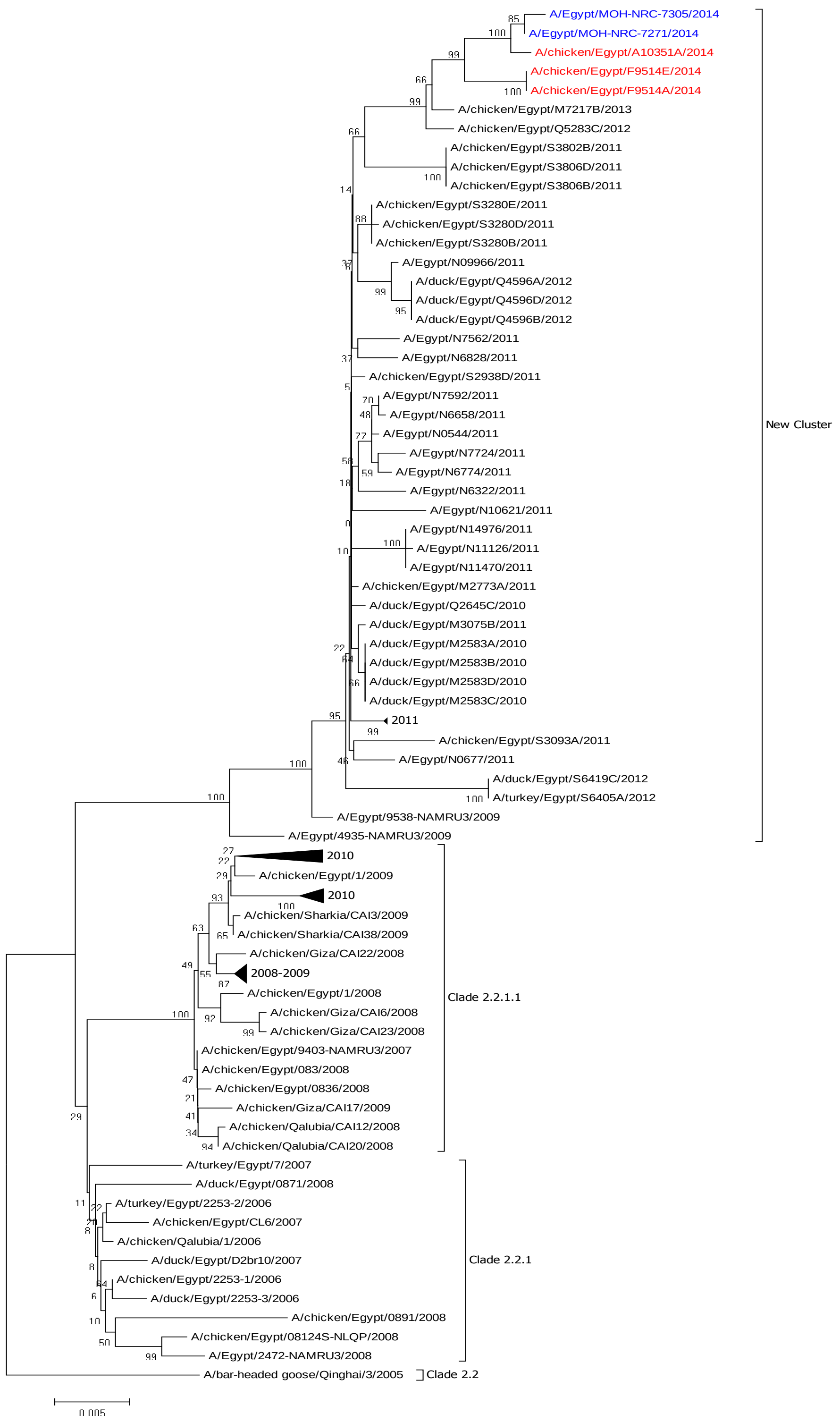

Technical Appendix Figure 2. Phylogenetic tree of the PB1 gene. Phylogenetic analysis was done using the neighbor-joining algorithm with the Kimura 2-parameter model. A/bar-headed goose/Qinghai/3/2005 was used as the root for the tree and the reliability of phylogenetic inference at each branch node was estimated by the bootstrap method with 1,000 replications, evolutionary analysis were conducted in MEGA6. Human viruses sequenced for this study are marked with a blue color. Avian viruses were isolated in 2014 and sequenced for this paper are marked with a red color.

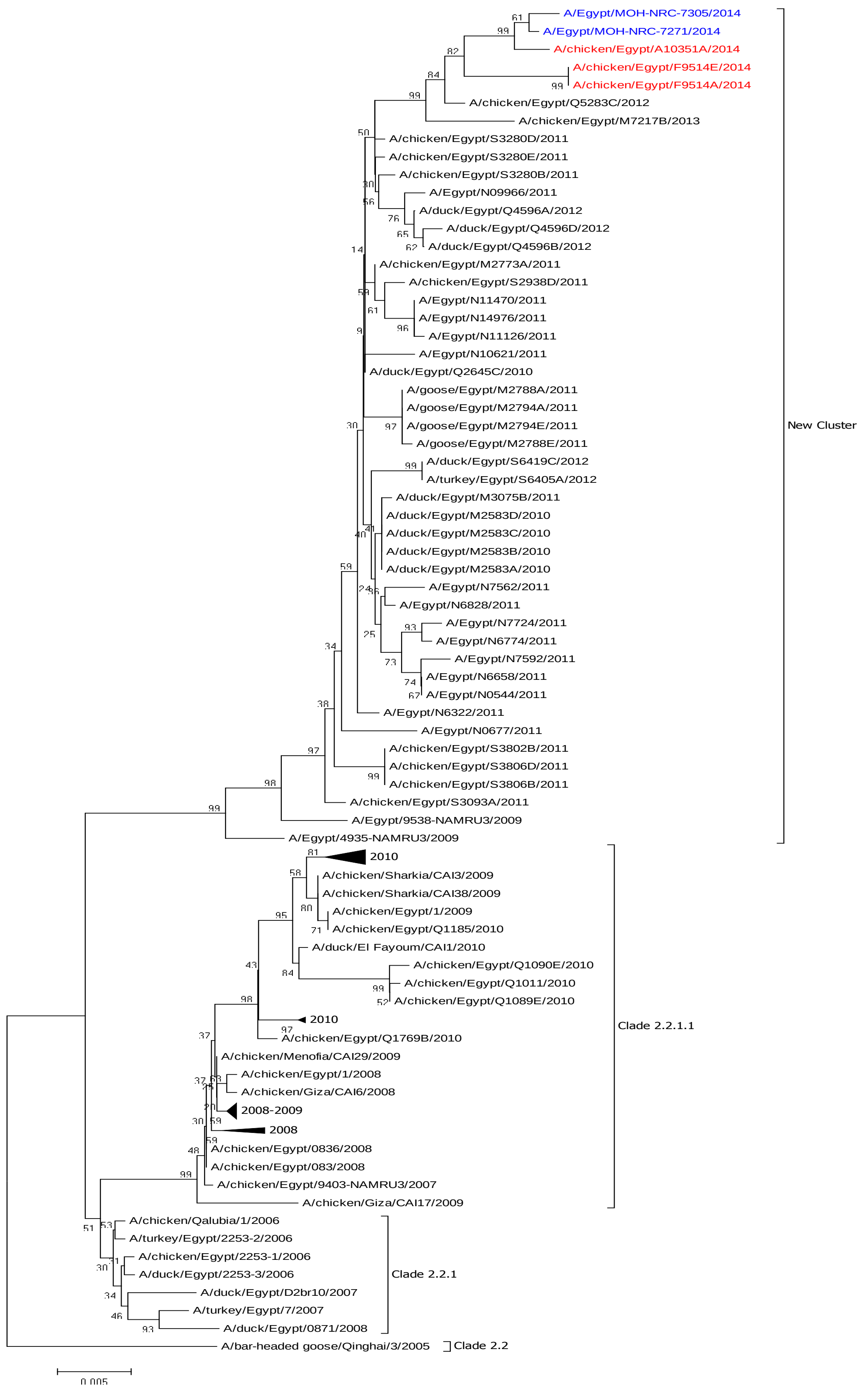

Technical Appendix Figure 3. Phylogenetic tree of the PA gene. Phylogenetic analysis was done using the neighbor-joining algorithm with the Kimura 2-parameter model. A/bar-headed goose/Qinghai/3/2005 was used as the root for the tree and the reliability of phylogenetic inference at each branch node was estimated by the bootstrap method with 1,000 replications, evolutionary analysis were conducted in MEGA6. Human viruses sequenced for this study are marked with a blue color. Avian viruses were isolated in 2014 and sequenced for this paper are marked with a red color.

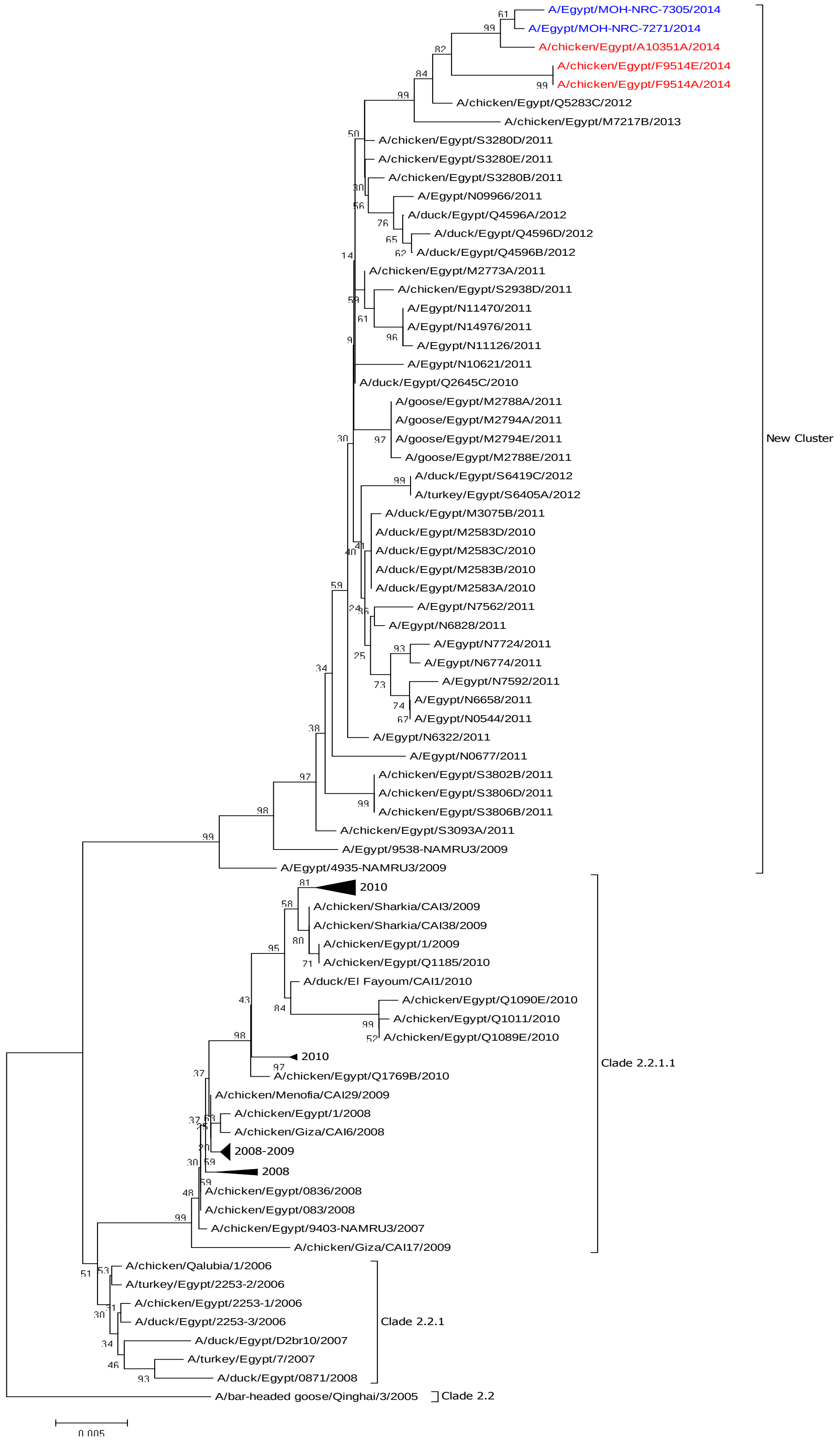

Technical Appendix Figure 4. Phylogenetic tree of the NP gene. Phylogenetic analysis was done using the neighbor-joining algorithm with the Kimura 2-parameter model. A/bar-headed goose/Qinghai/3/2005 was used as the root for the tree and the reliability of phylogenetic inference at each branch node was estimated by the bootstrap method with 1,000 replications, evolutionary analysis were conducted in MEGA6. Human viruses sequenced for this study are marked with a blue color. Avian viruses were isolated in 2014 and sequenced for this paper are marked with a red color.

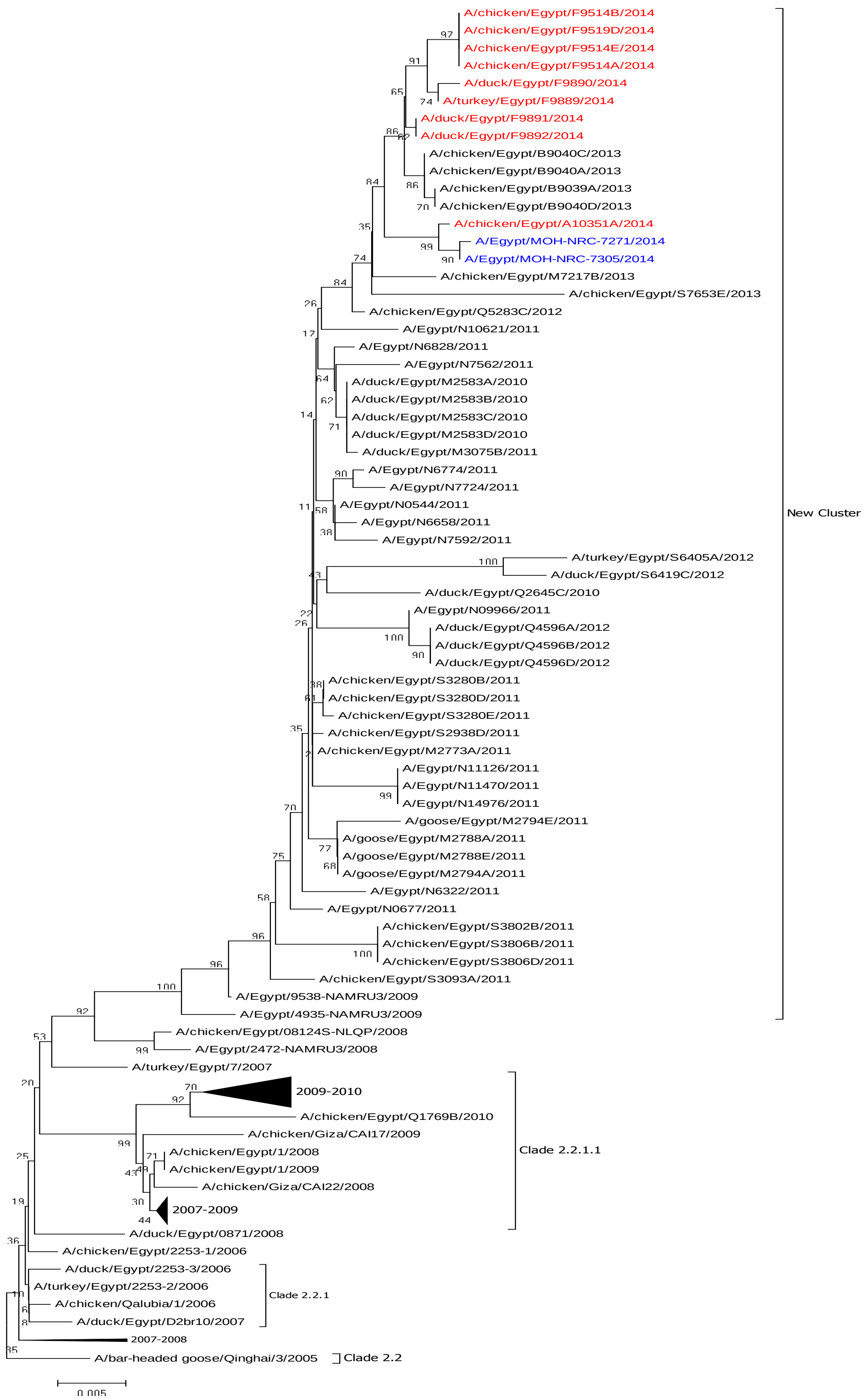

Technical Appendix Figure 5. Phylogenetic tree of the NA gene. Phylogenetic analysis was done using the neighbor-joining algorithm with the Kimura 2-parameter model. A/bar-headed goose/Qinghai/3/2005 was used as the root for the tree and the reliability of phylogenetic inference at each branch node was estimated by the bootstrap method with 1,000 replications, evolutionary analysis were conducted in MEGA6. Human viruses sequenced for this study are marked with a blue color. Avian viruses were isolated in 2014 and sequenced for this paper are marked with a red color.

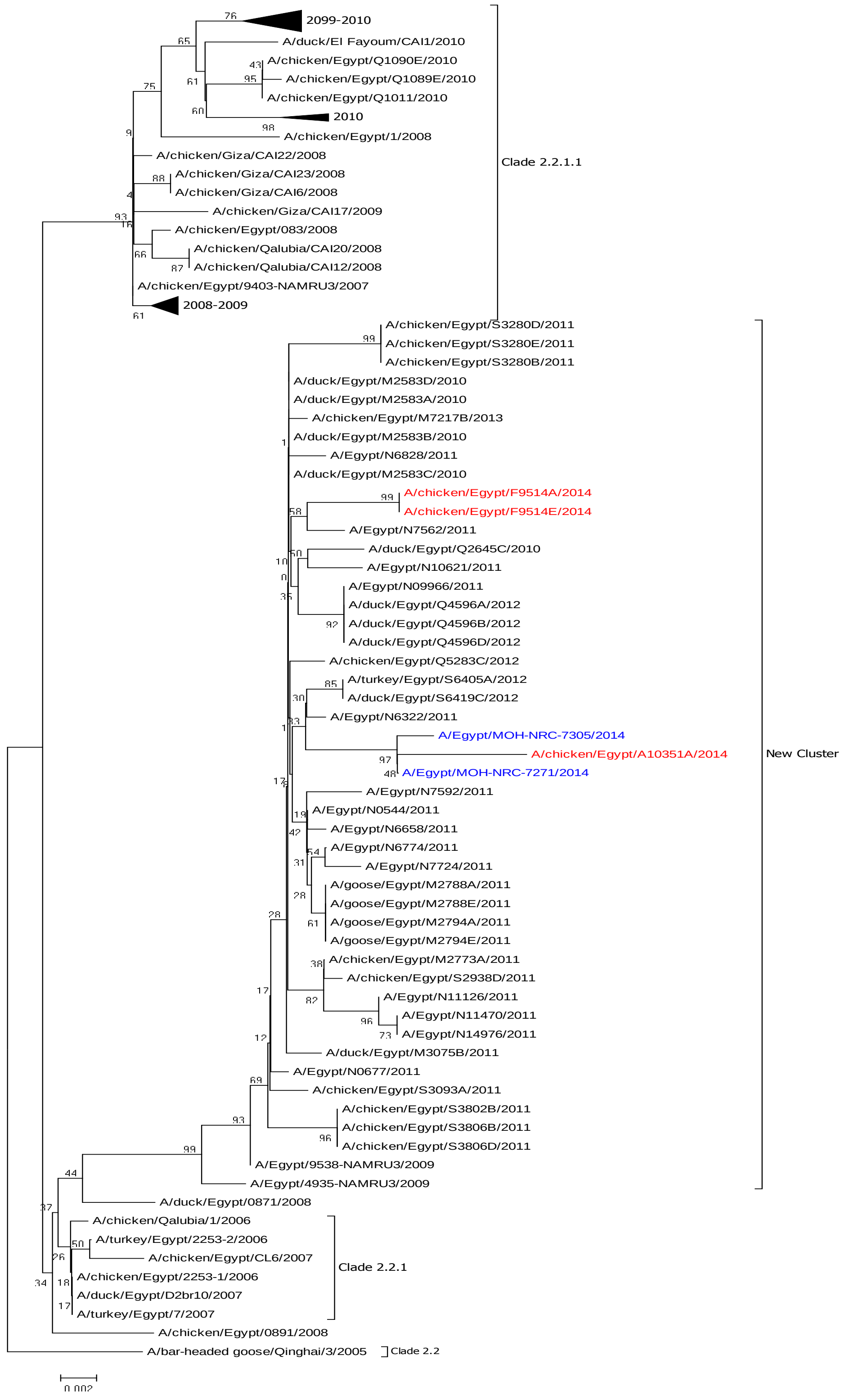

Technical Appendix Figure 6. Phylogenetic tree of the M gene. Phylogenetic analysis was done using the neighbor-joining algorithm with the Kimura 2-parameter model. A/bar-headed goose/Qinghai/3/2005 was used as the root for the tree and the reliability of phylogenetic inference at each branch node was estimated by the bootstrap method with 1,000 replications, evolutionary analysis were conducted in MEGA6. Human viruses sequenced for this study are marked with a blue color. Avian viruses were isolated in 2014 and sequenced for this paper are marked with a red color.

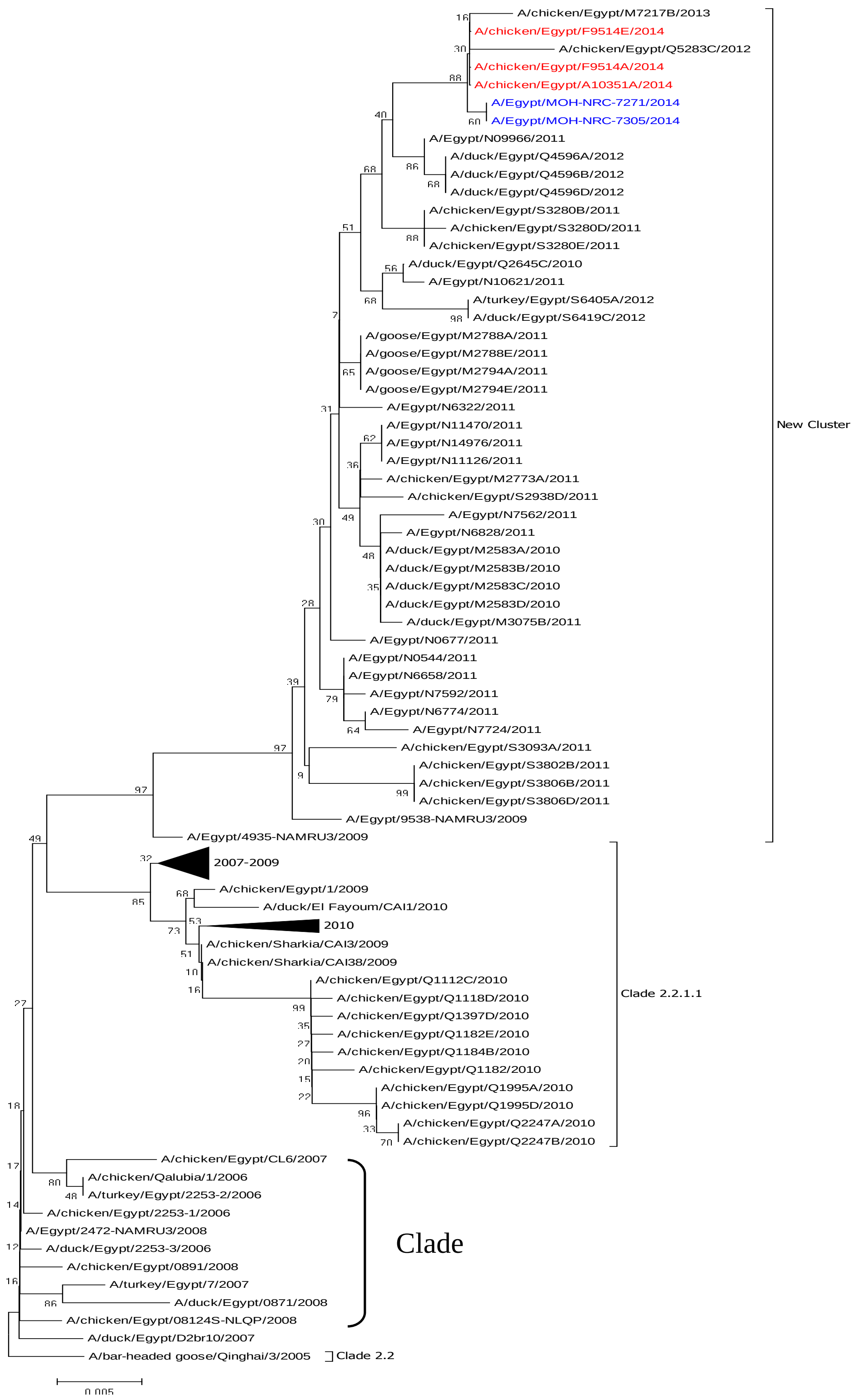

Technical Appendix Figure 7. Phylogenetic tree of the NS gene. Phylogenetic analysis was done using the neighbor-joining algorithm with the Kimura 2-parameter model. A/bar-headed goose/Qinghai/3/2005 was used as the root for the tree and the reliability of phylogenetic inference at each branch node was estimated by the bootstrap method with 1,000 replications, evolutionary analysis were conducted in MEGA6. Human viruses sequenced for this study are marked with a blue color. Avian viruses were isolated in 2014 and sequenced for this paper are marked with a red color.
